# Myocardial Injury Caused by Chronic Alcohol Exposure—A Pilot Study Based on Proteomics

**DOI:** 10.3390/molecules27134284

**Published:** 2022-07-03

**Authors:** Xiaonan Ma, Zihan Liao, Rongxuan Li, Wei Xia, Honghui Guo, Jiawei Luo, Huaxin Sheng, Meihui Tian, Zhipeng Cao

**Affiliations:** 1 Department of Forensic Pathology, School of Forensic Medicine, China Medical University, Shenyang 110122, China; mydjx929@foxmail.com (X.M.); zihanliao@hotmail.com (Z.L.); lirxdoctor@126.com (R.L.); ljw20210903@outlook.com (J.L.); 2 The Third Clinical Department, China Medical University, Shenyang 110122, China; 3 Liaoning Province Key Laboratory of Forensic Bio-Evidence Science, Shenyang 110122, China; 4 Department of Forensic Analytical Toxicology, School of Forensic Medicine, China Medical University, Shenyang 110122, China; lessenziale123@outlook.com (W.X.); 18856834146@163.com (H.G.); 5 Multidisciplinary Neuroprotection Laboratories, Center of Perioperative Organ Protection, Department of Anesthesiology, Duke University Medical Center, Durham, NC 27710, USA; sheng001@mc.duke.edu; 6 Department of Forensic Genetics and Biology, School of Forensic Medicine, China Medical University, Shenyang 110122, China

**Keywords:** alcoholic cardiomyopathy, proteomics, differentially expressed proteins, KEGG pathway, myocardial injury, alcohol exposure

## Abstract

Chronic alcohol exposure can cause myocardial degenerative diseases, manifested as cardiac insufficiency, arrhythmia, etc. These are defined as alcoholic cardiomyopathy (ACM). Alcohol-mediated myocardial injury has previously been studied through metabolomics, and it has been proved to be involved in the Kyoto Encyclopedia of Genes and Genomes (KEGG) pathway concerning unsaturated fatty acids biosynthesis and oxidative phosphorylation, which tentatively explored the mechanism of ACM induced by chronic drinking. To further study alcohol-induced myocardial injury, myocardial specimens from a previously successfully established mouse model of ACM were subjected to histological, echocardiographic, and proteomic analyses, and validated by real-time quantitative polymerase chain reaction (qPCR). Results of histopathology and echocardiography showed the hypertrophy of cardiomyocytes, the dilation of ventricles, and decreased cardiac function. Proteomic results, available via ProteomeXchange with identifier PXD032949, revealed 56 differentially expressed proteins (DEPs) were identified, which have the potential to be involved in the KEGG pathway related to fatty acid biosynthesis disorders, lipid metabolism disorders, oxidative stress, and, ultimately, in the development of dilated cardiomyopathy (DCM). The present study further elucidates the underlying effects of myocardial injury due to chronic alcohol intake, laying a foundation for further studies to clarify the potential mechanisms of ACM.

## 1. Introduction

The advancement of social economy and the progress of quality of life has brought a series of health problems, among which, the problem of excessive drinking has aroused widespread concern in society [1]. Long-term or excessive drinking can cause a series of health problems, including alcoholic cardiomyopathy (ACM). The incidence of alcoholic cardiomyopathy has been increasing year by year, and it has become an increasingly prevalent disease in the modern cardiovascular field. According to the World Health Organization, alcohol causes approximately 4% of all deaths each year worldwide, making it a major problem for human health [2]. In developed countries, ACM is the main cause of left ventricular dysfunction. In the US, ACM ranks first in non-ischemic cardiomyopathy deaths. Epidemiological data suggest that ACM accounts for 3.8% to 47% of non-ischemic cardiomyopathy [3,4]. Although many studies have been devoted to ACM, its pathogenesis remains to be further explored.

The toxic effects of chronic alcoholism on the heart are mainly divided into two aspects: the comprehensive effects of alcohol and its metabolites and chronic malnutrition. They can cause myocardial degeneration, which leads to ACM with dilation of the ventricle, cardiac insufficiency, and arrhythmia as the main manifestations. According to statistics, excessive alcohol consumption can attribute to up to 40% of causes of dilated cardiomyopathy (DCM) [4].

Proteomics, the total protein content of organisms, tissues, or cells, is an important means to understand gene function [5]. As an effector of biological functions, the level of protein depends not only on the level of upstream mRNA, but also on the translational regulation of upstream signals. Through the qualitative, quantitative, molecular function analysis, pathway interaction analysis, and protein interaction analysis of the proteome, it reveals the biological function, mechanism of action. Therefore, proteomics is regarded as the most direct evidence reflecting biological functions, and it has a strong hint for the exploration of the pathogenesis [6]. Therefore, the present study aimed to explore the underlying mechanisms of myocardial injury caused by chronic alcohol exposure by employing histopathology, echocardiography, molecular biology, and proteomics methods.

## 2. Materials and Methods

### 2.1. Animal Model and Sample Collection

All animal experiments were performed under the review and supervision of the Animal Care and Use Committee of China Medical University (No. CMU2019266), and in accordance with the “Guidelines for the Care and Use of Laboratory Animals” (NIH Publication No. 86–23, Revised 1985).

The present experiment used the same batch of animal models as previous study [7]. Briefly, a total of sixteen 7-week-old male C57BL/6 mice (purchased from Beijing Vital River Laboratory Animal Technology Co., Ltd., Beijing, China) of SPF-grade were included in the experiment, and all the mice (8-week-old male) were adaptively raised for one week before fed with alcoholic diet. Then, the mice were divided into two groups randomly. The classic Lieber-DeCarli liquid diet was used to establish ACM model according to previous studies [8,9,10,11,12,13]. As the manufacturer’s instruction, mice in the experimental group (*n* = 8) were acclimated to the alcoholic liquid diet via a gradient approach of alcohol for one week, and then they were fed with 4% alcohol Lieber-DeCarli liquid diet (TP 4030B) for formal experiments. Mice in the control group (*n* = 8) were fed with alcohol-free liquid diet (TP 4030C). The same energy intake and other conditions between the two groups were strictly controlled.

After 12 weeks, the establishment of the ACM model was assessed by echocardiography, then the mice were sacrificed by cervical dislocation. Five mice in each group were used for histology studies and real-time quantitative polymerase chain reaction (qPCR). Myocardial tissue samples were taken from another three mice in each group, washed with cold phosphate-balanced solution (PBS), and frozen at −80 °C for proteomics analysis after liquid absorption quickly.

### 2.2. Ultrasonic Examination

Mice were anesthetized in a closed transparent box, followed by a face mask to maintain anesthesia. Ultrasonography was performed using a Vevo 2100 system and a 40 MHz mouse transducer (VisualSonics, Toronto, ON, Canada) to determine end-diastolic and end-systolic left ventricular wall thickness, left ventricular volume, left ventricular diameter, left ventricular ejection fraction, and fractional shortening.

### 2.3. Hematoxylin and Eosin (H&E) Staining

After being fixed in 4% paraformaldehyde for 24 h, washed with PBS, embedded in paraffin, dehydrated with gradient alcohol, and then dealcoholized in xylene, the heart specimens were finally cut into about 5 μm sections. These tissues were stained with standard H&E staining methods as provided directions [7].

### 2.4. Total Protein Extraction and Protein Quality Test

Myocardial tissue samples were ground, lysed, centrifuged, and the supernatant was extracted and incubated with acetone for 2 h. The collected precipitate was dissolved, and bovine serum albumin (BSA) solution (0 to 0.5 g/L) and sample solution at various dilutions were added. Quickly, 180 μL of G250 dye solution was added to detect and calculate the protein concentration of the sample. Protein samples were subjected to 12% SDS-PAGE gel electrophoresis. Gels were stained with Coomassie Brilliant Blue R-250 and decolored until bands were clearly visible.

### 2.5. Tandem Mass Tagging (TMT) Labelling of Peptides

Samples were diluted in lysis buffer and mixed with trypsin and TEAB buffer. After 4 h of digestion, trypsin and CaCl_2_ were added overnight. Then, after adjusting the pH with formic acid, the supernatant was slowly added to a C18 desalting column for washing and elution. The eluate was collected, lyophilized, and TEAB buffer and TMT labeling reagent (Thermo Fisher, Waltham, MA, USA) were added.

### 2.6. Separation of Fractions

Mobile phase liquid A (2% acetonitrile and ammonium hydroxide at pH = 10.0) was prepared. The mixed lyophilized powder was dissolved in liquid A and centrifuged. Elution was performed using an L-3000 HPLC system (Thermo Fisher, Waltham, MA, USA) with a Waters BEH C18 (4.6 × 250 mm, 5 μm) column set (Milford, MA, USA) at 45 °C (Table S1, Supplementary Materials). After freeze-drying, 0.1% formic acid was added to dissolve.

### 2.7. LC-MS/MS Analysis

First mobile phase liquid A (100% water, 0.1% formic acid) and liquid B (80% acetonitrile, 0.1% formic acid) was prepared. Then, 1 μg supernatant of each fraction sample was used for liquid quality detection using EASY-nLCTM1200 UHPLC system (Thermo Fisher, Waltham, MA, USA). Finally, the raw data (.raw) of MS detection was obtained (Table S2, Supplementary Materials).

### 2.8. Data Analysis

#### 2.8.1. The Identification and Quantitation of Protein

The software Proteome Discoverer 2.4 (Thermo Fisher, Waltham, MA, USA) was used for database searching, spectroscopic peptide, and protein quantification with parameter settings in Table S3 (Supplementary Materials). Only peptides and proteins with fault detection rate (FDR) less than 1% were retained. Proteins that were quantitatively significantly different between experimental and control groups with *p* value < 0.05 and fold change (FC) > 1.2 (|log2FC| > 0.263) or <0.83 (|log2FC| < 0.269) were defined as differentially expressed proteins (DEPs).

#### 2.8.2. The Functional Analysis of Protein and DEPs

Interproscan software (EMBL-EBI, Hinxton, UK) was used to perform Gene Ontolog (GO) and InterPro (IPR) functional analyses. Plus, protein family and pathway analysis were performed on the identified proteins by Orthologous Group (COG) and Kyoto Encyclopedia of Genes and Genomes (KEGG) databases. Volcano plot analysis, cluster heatmap analysis, and pathway enrichment analysis were performed on GO, IPR, and KEGG for DEP analysis and prediction of possible protein–protein interactions (PPIs).

### 2.9. Real-Time qPCR

Real-time qPCR was conducted as our previous methods [7]. The upstream and downstream primer sequences are shown in Table S4. The relative amount of each index cDNA was calculated by the ΔΔCT method with GAPDH as the internal reference to analyze the expression level of mRNA.

## 3. Results

### 3.1. Successful Construction of the ACM Model

#### 3.1.1. Assessment of Cardiac Function

By comparing the echocardiographic results of the control group and the ACM group, it was found that the hearts of the mice were dilated and the cardiac function decreased under chronic ethanol exposure. Moreover, the thickness of the left ventricular anterior and posterior walls at end-systole, cardiac ejection fraction, and fractional shortening in the ACM group was significantly reduced. In addition, end-systolic left ventricular volume was significantly increased in the ACM group (Table 1).

#### 3.1.2. H&E Staining

**Figure 1:**
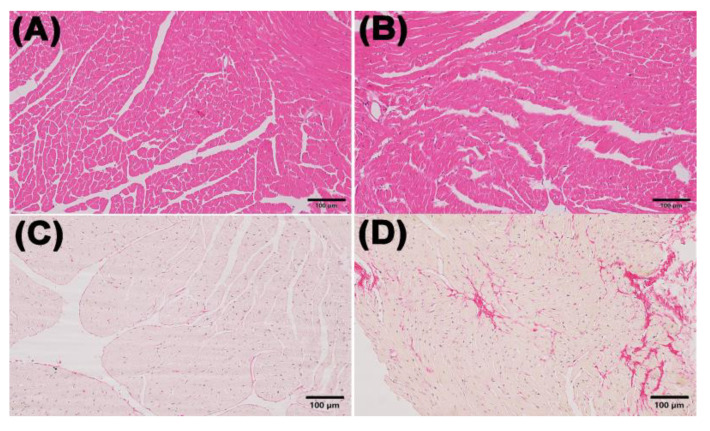
Morphological changes in the myocardium in the control and ACM group. (**A**) representative H&E staining for the control group (200×); (**B**) representative H&E staining for the ACM group (200×); (**C**) representative Sirius Red staining for the control group (200×); (**D**) representative Sirius Red staining for the ACM group (200×).

Myocardial H&E staining showed that the mice in the ACM group had hypertrophic cardiomyocytes and mildly dilated ventricles compared with the control group. The results of Sirius red staining showed myocardial fibrosis in mice of ACM group (Figure 1).

### 3.2. Proteomic Pattern in the Alcohol Exposure and Control Group

#### 3.2.1. Principal Component Analysis (PCA) Results

**Figure 2:**
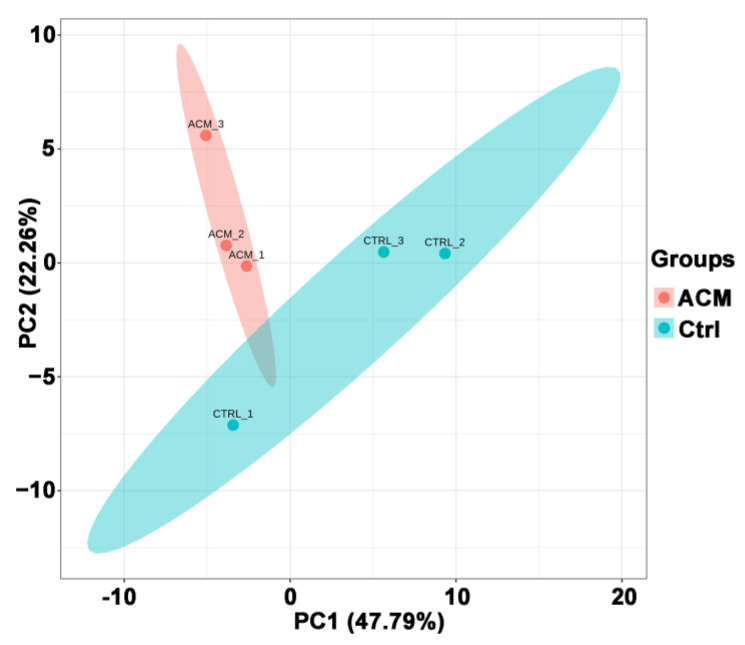
PCA principal component analysis between the control group and the ACM group.

PCA analysis showed a good separation between the ACM group and the control group (Figure 2). PC1 and PC2 represented 47.79% and 22.26%, respectively, of the total variability in the data set.

#### 3.2.2. Volcano Map and Heat Map

**Figure 3:**
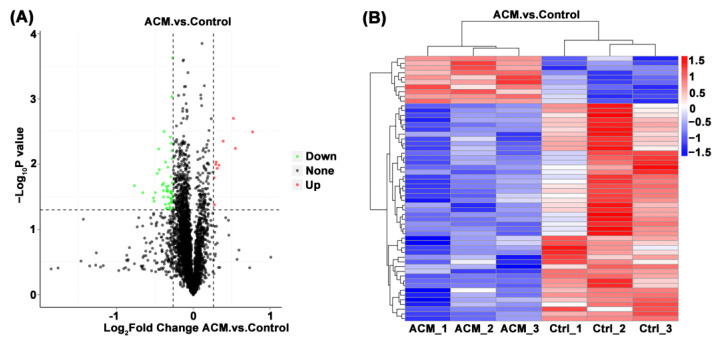
(**A**) Volcano plot of the myocardial proteomics data of the control group and the ACM group. DEPs are demonstrated in the volcano map. The abscissa is the logarithm of the protein difference multiple with 2 as the base, and the ordinate is the absolute value of the logarithm of the *p* value with 10 as the base. Black dots represent proteins that are not significantly different, and red and green represent up-regulated and down-regulated proteins, respectively. (**B**) Heat map of the differentially expressed metabolites between the control group and the ACM group. The up-regulation and down-regulation proteins among different samples are revealed by cluster analysis. Each row is adjusted for Z value, (observed value-row mean)/row standard deviation. Red and blue represent up-regulation and down-regulation, respectively.

Through the comparison between the ACM group and the control group, a total of 56 DEPs were screened, including 10 upregulated proteins and 46 downregulated proteins (Table 2 and Table 3). The volcano map and heat map are shown in Figure 3. When FC ≥ 1.2 and *p* value ≤ 0.05, the upregulated protein is screened out. When FC ≤ 0.83 and *p* value ≤ 0.05, the downregulated protein was screened out.

#### 3.2.3. Functional Enrichment Analysis

**Figure 4:**
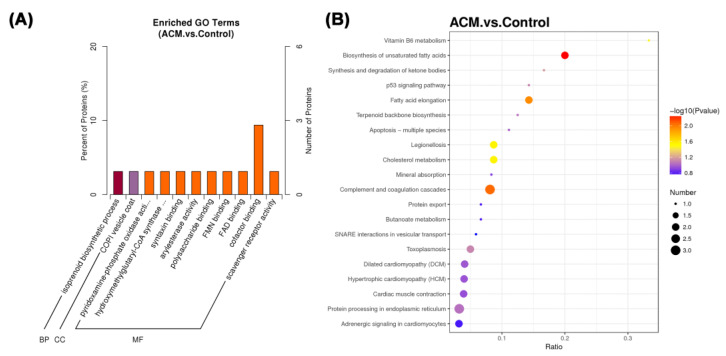
(**A**) Functional enrichment of GO annotation for DEPs. BP: biological process, CC: cellular component, and MF: molecular function. (**B**) The KEGG pathway enrichment analysis of different groups between the control group and the ACM group. The ratio of the number of differential proteins to the total number of proteins identified is the abscissa. From blue to red dots, it means that the adjusted *p* value is increasing from large to small, and the degree of enrichment is becoming more and more significant. The size of the dots represents the number of genes enriched in this pathway.

GO enrichment analysis was conducted to explore the functional characteristics and the degree of enrichment of DEPs (Figure 4A). The related biological process of the screened DEPs in ACM group was isoprenoid biosynthetic process in comparison with the control group. These DEPs are mainly located in the COPI vesicle coat and their main molecular functions were pyridoxamine-phosphate oxidase activity, hydroxymethylglutaryl-CoA synthase activity, syntaxin binding, arylesterase activity, etc.

The major KEGG pathway and related DEPs are demonstrated in Table 4. The main enriched pathways of the ACM group were biosynthesis of unsaturated fatty acids, fatty acid elongation, legionellosis, cholesterol metabolism, Vitamin B6 metabolism, etc. (Figure 4B).

#### 3.2.4. PPI Network Analysis

**Figure 5:**
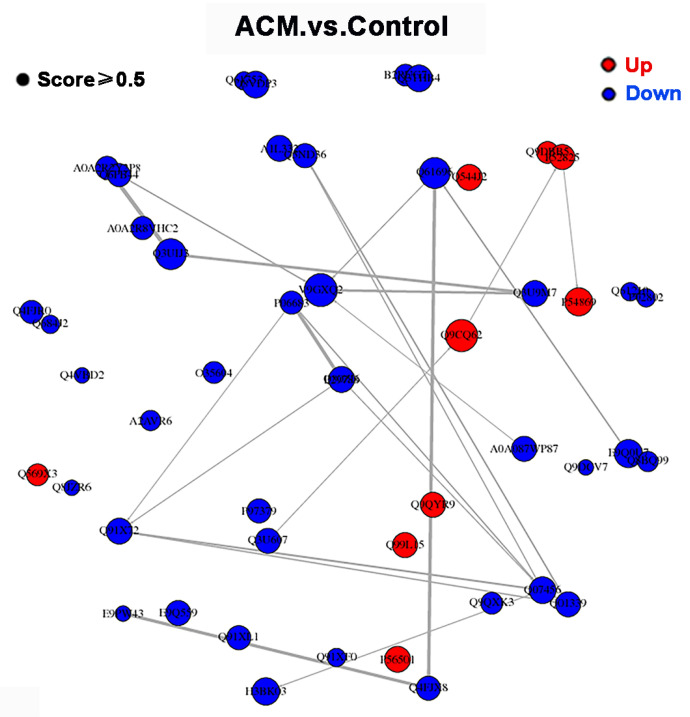
PPI network of DEPs between control group and ACM group.

The PPI network of DEPs between control group and ACM group is shown in Figure 5. However, the major nodal DEPs did not show close interaction between the two groups.

### 3.3. Real-Time qPCR Results

**Figure 6:**
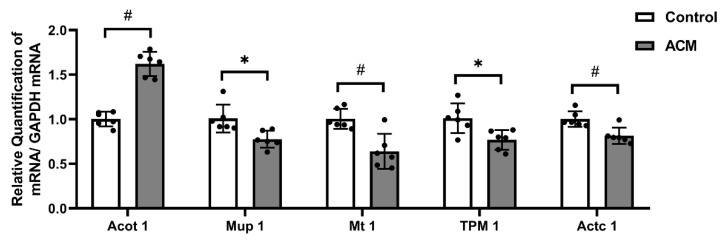
Real-time qPCR results of Acot 1, Mup 1, Mt 1, TPM 1, and Actc 1 mRNA (# *p* < 0.01; * *p* < 0.05).

Real-time qPCR results showed that the mRNA expression level of Acyl-coenzyme A thioesterase 1 (Acot 1) in the myocardial tissue of mice in the ACM group was significantly increased (*p* < 0.01), while the mRNA expression levels of major urinary protein 1 (Mup1) (*p* < 0.05), Metallothionein 1 (Mt 1) (*p* < 0.01), Tropomyosin 1 (TPM 1) (*p* < 0.05), and Alpha cardiac muscle 1 (Actc 1) (*p* < 0.01) were significantly decreased (Figure 6).

## 4. Discussion

With the improvement of the socioeconomic level, the health problems caused by long-term and excessive drinking have gradually attracted social attention. Relevant studies have shown the direct toxic effects of ethanol and its metabolites on the heart. Oxidative stress, fatty acid metabolism disorders, etc., may be the underlying mechanisms of alcohol injury to the myocardium [14,15]. In the present study, echocardiography confirmed cardiac dilation and decreased cardiac function in ACM mice. H&E staining was used to investigate the morphological changes of myocardial tissue in ACM model mice, suggesting that myocardial cell hypertrophy and structural disorders. In the proteomic study, 56 DEPs were screened and KEGG enrichment analysis showed they were related to fatty acid biosynthesis, lipid metabolism, oxidative stress, ventricular dilatation, development of DCM, and so on. The major DEPs and their related KEGG pathways will be further discussed below.

Alcohol can affect the utilization of fatty acids by cardiomyocytes, which may be achieved by affecting the absorption of long-chain fatty acids, the accumulation of triglycerides and phosphatidyl alcohols, etc. [16]. Studies have found that Acot 1 protein is involved in unsaturated fatty acid synthesis and fatty acid chain elongation [17]. Acot 1 is a member of the Acots family that hydrolyze acyl-CoA into free fatty acids and CoA, maintaining free fatty acids and acyl-CoA, which plays an important role in fatty acid metabolism [18,19]. Fujita et al. found that the expression of Acot 1 in cardiomyocytes in a rodent model of high-fat diet increased significantly, suggesting that the overexpression of Acot 1 will accelerate the differentiation of mature adipocytes and excessive lipid accumulation [20,21,22]. In the present study, long-term exposure to ethanol produced a similar effect, with Acot 1 mRNA and protein significantly increased. It is suggested that fatty acid metabolism disorder may be one of the potential mechanisms of myocardial injury caused by chronic alcohol exposure, which is consistent with previous metabolomics results [7].

The correlation between abnormal lipid metabolism and the development of cardiovascular disease has been widely reported [23,24]. Chronic ethanol exposure leads to marked increases in plasma cholesterol and triglycerides, which in turn lead to myocardial injury [25]. Studies have shown that Apolipoprotein H (Apo H) can inhibit the accumulation of intracellular cholesterol, and low expression of Apo H is associated with elevated blood lipid levels and atherosclerosis [26,27,28,29]. In addition, Mup 1 belongs to the lipocalin family and is mainly expressed in liver and skeletal muscle. Low levels of Mup 1 protein expression result in increased lipid levels in blood lipids, liver, and skeletal muscle [30,31]. To further corroborate the metabolomic study, the present study found that Apo H and Mup 1 levels related to lipid metabolism were down-regulated in the ACM group, which may be related to myocardial injury induced by chronic ethanol exposure [7].

Oxidative stress can occur when the content of reactive oxygen species is increased due to hypoxia, and it is an important factor in causing myocardial damage. Numerous studies have confirmed that both acute and chronic alcohol exposure led to an increase in oxidants and a decrease in antioxidants, suggesting that oxidative stress may be involved in the development of ACM [32,33]. Mt 1 is a metal-binding protein with powerful antioxidant properties. Studies have confirmed that it exerts significant impacts in scavenging free radicals, protecting nucleic acids against toxic damage, and improving tissue inflammation [34,35,36]. The present study showed that the expression of Mt 1 mRNA and protein in cardiomyocytes in the ACM group was significantly decreased, significantly weakening its effect on myocardial protection through antioxidants. In addition, paraoxonase 1 (Pon 1) is an antioxidant enzyme that has been shown to protect tissues from oxidative damage and lipid peroxidation [37]. It is also present in high-density lipoprotein (HDL), conferring antioxidant and anti-cholesterol properties to HDL and inhibiting the formation of oxidized low-density lipoprotein [38,39,40,41]. The downregulation of Pon 1 expression in the ACM group in the present study suggests that oxidative stress is associated with alcohol-induced myocardial injury. At the same time, the expression of Acot 1 is also increased in the ACM group, and its increased expression can reduce oxidative stress and improve myocardial function [17].

ACM is characterized by ventricular dilatation, arrhythmias, and cardiac insufficiency, which is one of the main reasons of non-ischemic DCM in the developed world [42,43]. GO enrichment and KEGG enrichment analysis found that some DEPs, mainly including TPM 1 and Actc 1 were associated with DCM and myocardial contraction. Studies have found that downregulation of TPM 1 and Actc 1 is closely related to muscle fiber contractile activity and muscle tissue development, which is consistent with the present findings [44,45]. TPM 1 is an isoform of tropomyosin [46]. Previous studies have identified that elevated levels of TPM 1 protein in myofilaments can lead to reduced ejection fraction, systolic and diastolic dysfunction, and reduced myofilament calcium sensitivity, which play an important role in the development of DCM [47]. Mazzarotto et al. demonstrated that variants of TPM 1 and Actc 1 were found to be significantly enriched in selected DCM patients through clinical studies [48]. Detection of Actc 1 mutations has clinical implications for monitoring delayed DCM [49]. Therefore, TPM 1 and Actc 1 may be involved in the process of DCM induced by alcohol exposure, but the specific mechanism still needs to be further studied. In addition, numerous studies have shown that Ring finger protein 2 (Rnf 2) is important for maintaining normal cardiac systolic function. Rnf 2 deficiency disrupts atrioventricular canal and sinoatrial node construction by regulating the expression atrioventricular canal marker genes, resulting in impaired cardiac conduction system and further cardiac systolic dysfunction [50,51].

There are still some limitations in the present study. First, the number of animals included in the proteomics was relatively small. Second, the screened DEPs were not validated by western blotting or immunohistology (IHC) staining. According to previous reports, the establishment of the mouse model of ACM in the present study was performed with 8-week-old mice [10,52]. Nonetheless, according to instructions of The Jackson Laboratory (www.jax.org, accessed on 24 June 2022), 8-week-old mice may be immature and may have some influence on the results of this study, which is also a limitation of this paper. Therefore, the present study is just a pilot study on myocardial injury caused by chronic ethanol exposure providing the result of proteomics which should be further explored in the future. In addition, the use of alcoholic cardiomyopathy models in humans and the impact of genetic differences between species on research should also be considered. Machine learning may have the potential to provide solutions to our problems.

## 5. Conclusions

In conclusion, this study employed histology, echocardiography, proteomics, and real-time qPCR to investigate myocardial injury induced by chronic ethanol exposure which induced the hypertrophy of cardiomyocytes, the dilation of ventricle and decreased cardiac function. Proteomic results revealed chronic ethanol consumption can induce fatty acid and lipid metabolism disorders as well as oxidative stress in cardiomyocytes, which are ultimately involved in the occurrence and development of DCM. The study explored the causes of myocardial injury caused by chronic alcohol exposure from the perspective of proteomics, which highlights the direction for further study of its mechanism and lays a foundation for further studies, especially translation on humans in the future.

## Figures and Tables

**Table 1:** The results of echocardiography.

Parameters	Control Group	ACM Group
LVAW; d (mm)	0.866 ± 0.069	0.599 ± 0.094 #
LVAW; s (mm)	0.772 ± 0.106	0.456 ± 0.053 *
LVPW; d (mm)	0.820 ± 0.096	0.619 ± 0.098
LVPW; s (mm)	0.899 ± 0.046	0.565 ± 0.110 *
LVID; d (mm)	4.721 ± 0.654	6.490 ± 0.587 #
LVID; s (mm)	4.089 ± 0.818	5.606 ± 0.788
LV Vol; d (μL)	79.760 ± 5.867	90.470 ± 2.809 #
LV Vol; s (μL)	41.804 ± 3.617	57.553 ± 3.571 *
EF%	51.442 ± 2.220	38.350 ± 1.925 *
FS%	24.924 ± 1.813	19.290 ± 0.848 *

# *p* < 0.01; * *p* <0.05. (EF%: ejection fraction, FS%: fractional shortening, LVPW; d: left ventricular posterior diastolic wall thickness, LVPW; s: left ventricular posterior systolic wall thickness, LVAW; d: left ventricular anterior diastolic wall thickness, LVAW; s: left ventricular anterior systolic wall thickness, LVID; d: left ventricular end-diastolic diameter, LVID;s: left ventricular end-systolic diameter, LV Vol; d: left ventricular end-diastolic volume, and LV Vol; s: left ventricular end-systolic volume).

**Table 2:** The up-trend DEPs between the ACM group and the control group.

Protein	Description	Gene	Fold Change	*p* Value	Trend
Q544J2	Protein-serine/threonine kinase	Pdk4	1.436	0.002	up
P54869	Hydroxymethylglutaryl-CoA synthase, mitochondrial	Hmgcs2	1.709	0.003	up
Q569X3	U1 small nuclear ribonucleoprotein C	Snrpc	1.310	0.004	up
Q99L15	Acot1 protein (Fragment)	Acot1	1.463	0.006	up
Q9QYR9	Acyl-coenzyme A thioesterase 2, mitochondrial	Acot2	1.230	0.009	up
P52825	Carnitine O-palmitoyltransferase 2, mitochondrial	Cpt2	1.225	0.010	up
Q8R370	Usher syndrome type-1C protein-binding protein 1	Ushbp1	1.261	0.010	up
Q9CQ62	2,4-dienoyl-CoA reductase [(3E)-enoyl-CoA-producing], mitochondrial	Decr1	1.241	0.012	up
Q9DBB5	Eukaryotic translation initiation factor 4E type 3	Eif4e3	1.208	0.016	up
P56501	Mitochondrial uncoupling protein 3	Ucp3	1.211	0.042	up

**Table 3:** The down-trend DEPs between the ACM group and the control group.

Protein	Description	Gene	Fold Change	*p* Value	Trend
Q4FJX8	Proteasome subunit beta	Psmb10	0.829	0.0002	down
A0A087WP87	RING-type E3 ubiquitin transferase (Fragment)	Rnf2	0.824	0.001	down
Q61210	Rho guanine nucleotide exchange factor 1	Arhgef1	0.767	0.003	down
A2ADZ4	Alpha-taxilin (Fragment)	Txlna	0.813	0.004	down
Q3U607	Uncharacterized protein	Casp8	0.820	0.005	down
Q91XL1	Leucine-rich HEV glycoprotein	Lrg1	0.732	0.006	down
Q684J2	Serine/threonine kinase 23, muscle-specific serine kinase 1 70 (Fragment)	Srpk3	0.825	0.006	down
Q3U9M7	Uncharacterized protein	Lcp1	0.827	0.008	down
Q4FZE8	Major urinary protein 1	Mup22	0.779	0.009	down
Q8BQ99	Uncharacterized protein	Mrps2	0.795	0.011	down
Q4VBD2	Transmembrane anterior posterior transformation protein 1	Tapt1	0.736	0.012	down
P02802	Metallothionein-1	Mt1	0.723	0.014	down
H3BKQ2	Intraflagellar transport-associated protein (Fragment)	Iftap	0.817	0.016	down
B2RUG7	Zinc finger RNA binding protein	Zfr	0.826	0.019	down
E9Q559	Calcium-transporting ATPase	Atp2a3	0.764	0.020	down
A2AVR6	Acyl-coenzyme A thioesterase 11	Acot11	0.757	0.021	down
A2CEL1	Major urinary protein 1	Mup1	0.588	0.021	down
Q8JZR6	Electroneutral sodium bicarbonate exchanger 1	Slc4a8	0.799	0.022	down
A0A2R2Y2P8	Tropomyosin 1 kappa	Tpm1	0.785	0.022	down
P06683	Complement component C9	C9	0.790	0.024	down
Q61696	Heat shock 70 kDa protein 1A	Hspa1a	0.764	0.025	down
Q4FJR0	Nudt4 protein	Nudt4	0.794	0.026	down
Q91XF0	Pyridoxine-5-phosphate oxidase	Pnpo	0.818	0.026	down
Q6PB44	Tyrosine-protein phosphatase non-receptor type 23	Ptpn23	0.790	0.026	down
Q07456	Protein AMBP	Ambp	0.819	0.027	down
Q3UIJ3	Uncharacterized protein	Actc1	0.634	0.027	down
V9GXQ2	Predicted gene 17087	Gm17087	0.710	0.027	down
Q61753	D-3-phosphoglycerate dehydrogenase	Phgdh	0.816	0.027	down
O35604	NPC intracellular cholesterol transporter 1	Npc1	0.824	0.029	down
Q8VDP3	[F-actin]-monooxygenase MICAL1	Mical1	0.832	0.032	down
Q3TX70	t-SNARE coiled-coil homology domain-containing protein	Stx6	0.764	0.032	down
A0A2R8VHC2	GTP-binding protein 1 (Fragment)	Gtpbp1	0.796	0.032	down
H3BK03	Serum paraoxonase/arylesterase 1 (Fragment)	Pon1	0.699	0.033	down
Q9QXK3	Coatomer subunit gamma-2	Copg2	0.821	0.034	down
E9Q0U7	Heat shock protein 105 kDa	Hsph1	0.816	0.034	down
Q8VE86	Uncharacterized protein	Lypd8l	0.821	0.037	down
Q5ND36	Serine or cysteine peptidase inhibitor clade F member 2	Serpinf2	0.701	0.037	down
Q3THB4	L-lactate dehydrogenase	Ldha	0.822	0.038	down
P97379	Ras GTPase-activating protein-binding protein 2	G3bp2	0.821	0.039	down
Q80ZI6	E3 ubiquitin-protein ligase LRSAM1	Lrsam1	0.803	0.040	down
Q9DCV7	Keratin, type II cytoskeletal 7	Krt7	0.778	0.041	down
Q01339	Beta-2-glycoprotein 1	Apoh	0.809	0.045	down
E9PW43	Predicted pseudogene 10320	Gm10320	0.787	0.047	down
A1L332	Phospholipid-transporting ATPase	Atp8a1	0.829	0.048	down
P29788	Vitronectin	Vtn	0.811	0.048	down
Q91X72	Hemopexin	Hpx	0.786	0.048	down

**Table 4:** The major KEGG pathways and related DEPs.

Map Title	*p* Value	Description
Biosynthesis of unsaturated fatty acids	0.006	Acyl-coenzyme A thioesterase 2, mitochondrial, Acot1 protein (Fragment)
Complement and coagulation cascades	0.009	Vitronectin, Serine or cysteine peptidase inhibitor clade F member 2, Complement component C9
Fatty acid elongation	0.011	Acyl-coenzyme A thioesterase 2, mitochondrial, Acot1 protein (Fragment)
Cholesterol metabolism	0.029	Beta-2-glycoprotein 1, NPC intracellular cholesterol transporter 1
Legionellosis	0.029	Heat shock 70 kDa protein 1A, Uncharacterized protein
Vitamin B6 metabolism	0.035	Pyridoxine-5-phosphate oxidase
Synthesis and degradation of ketone bodies	0.069	Hydroxymethylglutaryl-CoA synthase, mitochondrial
Toxoplasmosis	0.079	Heat shock 70 kDa protein 1A, Uncharacterized protein
p53 signaling pathway	0.080	Uncharacterized protein
Terpenoid backbone biosynthesis	0.091	Hydroxymethylglutaryl-CoA synthase, mitochondrial
Protein processing in endoplasmic reticulum	0.094	Heat shock 70 kDa protein 1A, Heat shock protein 105 kDa, Predicted pseudogene 10320
Apoptosis—multiple species	0.101	Uncharacterized protein
Dilated cardiomyopathy (DCM)	0.112	Uncharacterized protein, Tropomyosin 1 kappa
Hypertrophic cardiomyopathy (HCM)	0.116	Uncharacterized protein, Tropomyosin 1 kappa
Cardiac muscle contraction	0.120	Uncharacterized protein, Tropomyosin 1 kappa
Mineral absorption	0.133	Metallothionein-1
Butanoate metabolism	0.164	Hydroxymethylglutaryl-CoA synthase, mitochondrial
Protein export	0.164	Predicted pseudogene 10320
Adrenergic signaling in cardiomyocytes	0.169	Uncharacterized protein, Tropomyosin 1 kappa
SNARE interactions in vesicular transport	0.183	t-SNARE coiled-coil homology domain-containing protein

## Data Availability

The mass spectrometry proteomics data have been deposited to the ProteomeXchange Consortium via the PRIDE partner repository with the dataset identifier PXD032949. The data presented in this study are available in the article and Supplementary Materials.

## Supplementary Materials

The following supporting information can be downloaded at: https://www.mdpi.com/article/10.3390/molecules27134284/s1, Table S1: Peptide fraction separation liquid chromatography elution gradient table; Table S2: Liquid chromatography elution gradient table; Table S3: The analysis parameter of Proteome Discoverer 2.4; Table S4: The primer sequences from NCBI.

## Sample Availability

Samples of the compounds are not available from the authors.
