# Supplementary material for: Myocardial Injury Caused by Chronic Alcohol Exposure—A Pilot Study Based on Proteomics
Source: Molecules. 2022 Jul 3;27(13):4284. doi: 10.3390/molecules27134284 (PMC9268295; doi:10.3390/molecules27134284)
Supplement: Supplementary file 1 [file molecules-27-04284-s001.zip › molecules-1763185-supplementary.pdf]

Supplementary tables

Table S1. Peptide fraction separation liquid chromatography elution gradient table.

Time (min)	Flow rate (mL/min)	Mobile phase A (%)	Mobile phase B (%)
0	1	97	3
10	1	95	5
30	1	80	20
48	1	60	40
50	1	50	50
53	1	30	70
54	1	0	100

Table S2. Liquid chromatography elution gradient table.

Time (min)	Flow rate (mL/min)	Mobile phase A (%)	Mobile phase B (%)
0	600	94	6
2	600	85	15
48.5	600	60	40
50.5	600	50	50
51.5	600	45	55
60	600	0	100

Table S3. The analysis parameter of Proteome Discoverer 2.4.

**Item**	**Value**
Type of Quantification	Reporter Quantification (TMT)
Enzyme	Trypsin
Max. Missed Cleavage Sites	2
Precursor Mass Tolerance	10 ppm
Fragment Mass Tolerance	0.02 Da
Dynamic Modification	Oxidation/+15.995 Da (M) and TMT /+229.163 Da (K)
N-Terminal Modification	Acetyl/+42.011 Da (N-Terminal) and TMT 10plex/+229.163 Da (K, Y) and Met-loss/-131.040Da(M) and Met-loss+Acetyl/-89.030 Da(M)
Static Modification	Carbamidomethyl/+57.021 Da (C)

Table S4. The primer sequences from NCBI.

Target	Species	Forward	Reverse
Acot 1	Mouse	AGTGCTGATTCAAGGGCTGG	TTCTCGCAGCTGGATTGAAC
Mup 1	Mouse	CCAGGCCCGAGAATGAAGAA	GGCCTCTCCATGAACACCAA
Mt 1	Mouse	CGACTTCAACGTCCTGAGTAC	GTCCATTCCGAGATCTGGTG
TPM 1	Mouse	ACAGAACCATTAACAGCCAGAG	TGAACAAAAGACCCCAGAGG
Actc 1	Mouse	AGGATGACCGTATTGTGCTTC	AACAAACTGCACTTGTGTAAACA
